# Distribution and Prevalence of Ticks and Tick-Borne Pathogens at the Wildlife-Livestock Interface in Africa: A Systematic Review

**DOI:** 10.3390/vetsci12040364

**Published:** 2025-04-13

**Authors:** Tsireledzo Goodwill Makwarela, Nimmi Seoraj-Pillai, Tshifhiwa Constance Nangammbi

**Affiliations:** Department of Nature Conservation, Faculty of Science, Tshwane University of Technology, Staatsartillerie Rd, Pretoria West, Pretoria 0183, South Africa; seorajpillayn@tut.ac.za (N.S.-P.); nangammbitc@tut.ac.za (T.C.N.)

**Keywords:** vector ecology, zoonotic pathogens, host–parasite interactions, cross-species transmission, rhipicephaline ticks, disease surveillance, One Health approach

## Abstract

Ticks and tick-borne diseases (TBDs) significantly impact African animal health and agricultural productivity, especially at the wildlife–livestock interface. This systematic review analyzed 20 eligible studies from East, Southern, and limited parts of Central Africa to determine the distribution and prevalence of key tick species and associated pathogens. *Rhipicephalus appendiculatus* and *Amblyomma variegatum* were the most commonly reported tick species, with *R. appendiculatus* exhibiting up to 50.5% prevalence in cattle and buffalo in Uganda. The most frequently detected pathogens included *Theileria parva*, *Anaplasma marginale*, and *Coxiella burnetii*, with *T. parva* showing high prevalence in cattle populations coexisting with wildlife. Notably, geographic disparities were observed, with Central and West Africa being underrepresented. Most pathogen detections occurred in Kenya, Uganda, Tanzania, Botswana, and South Africa, indicating regional hotspots for tick-borne disease transmission. This review highlights the urgent need for enhanced surveillance, region-specific vector control programs, and integrated One Health approaches to address the ecological, agricultural, and zoonotic challenges of tick-borne pathogens across Africa.

## Simple Summary

Ticks and the diseases they spread pose a growing threat to livestock and wildlife across Africa, particularly in areas where these animals interact. These diseases, transmitted by ticks such as *Rhipicephalus appendiculatus* and *Amblyomma variegatum*, reduce livestock productivity and endanger wildlife health, ultimately impacting farmers’ livelihoods and conservation efforts. This study systematically reviewed existing research to understand the distribution of ticks and the pathogens they carry at the wildlife-livestock interface. The findings highlight significant gaps in disease monitoring, particularly in Central and West Africa, where data is scarce. Our study emphasizes the importance of improved tick control measures, better disease surveillance, and integrating a One Health approach—considering the interconnected health of animals, humans, and the environment. Addressing these issues is critical to protecting economic and ecological stability in affected regions.

## 1. Introduction

Ticks are among the most significant vectors of disease-causing agents affecting both humans and animals [1,2]. As obligate hematophagous ectoparasites, ticks transmit a wide range of pathogens, including protozoa, bacteria, and viruses, which collectively cause a suite of tick-borne diseases (TBDs) [3,4,5]. In Africa, these diseases pose a major threat to livestock productivity, wildlife health, and, in some cases, human well-being [6,7,8]. Overlapping habitats and shared ecosystems characterize this interface, facilitating pathogen movement between wildlife reservoirs and domestic animals [9]. Wildlife presence often heightens the risk of tick infestations and TBD spread, as many wild species serve as reservoirs for pathogens that can infect livestock and humans [10,11,12]. The impact of ticks and TBDs is particularly critical at the wildlife–livestock interface, where increased contact between domestic and wild animals facilitates the exchange of vectors and pathogens across ecological boundaries [4,13,14].

Overlapping habitats and shared ecosystems characterize this interface, facilitating pathogen movement between wildlife reservoirs and domestic animals [9]. Wildlife presence often heightens the risk of tick infestations and TBD spread, as many wild species serve as reservoirs for pathogens that can infect livestock and humans [10,11,12]. Ticks and tick-borne diseases (TBDs) are significant challenges at the wildlife–livestock interface in Africa, where interactions among wildlife, livestock, and humans create an environment conducive to disease transmission [15]. The burden of tick-borne diseases (TBDs) is particularly pronounced at the wildlife–livestock interface, where contact between wild and domestic hosts increases opportunities for pathogen exchange and the emergence of new disease cycles [16,17]. Wildlife species often harbor various tick species and associated pathogens, which can spill over into livestock populations [18,19]. For instance, studies have documented *Rickettsia* and A. species in ticks collected from wildlife, highlighting a potential reservoir for these pathogens [20].

The increasing encroachment of human activities into natural ecosystems—driven by agricultural expansion, livestock production, and land fragmentation—has intensified interactions between wildlife and domestic animals [9,21]. Wildlife movements across landscapes can introduce new tick species and pathogens into livestock populations, complicating control efforts and heightening outbreak risks [12]. This interface has been recognized as a hotspot for disease emergence and transmission, where generalist tick vectors such as *Rhipicephalus appendiculatus* Neumann, 1901, *Amblyomma variegatum* (Fabricius, 1794), and *Hyalomma* spp. thrive across both wild and domestic hosts [22,23]. Consequently, pathogens such as *Theileria parva*, *Babesia bigemina*, *Anaplasma marginale*, and *Coxiella burnetii* are frequently detected in livestock populations that share grazing lands with wildlife [24,25,26]. Diseases such as *theileriosis* and *babesiosis*, transmitted by ticks, cause severe health issues in cattle, resulting in further economic losses [27]. The economic and veterinary implications of TBDs are profound. Losses due to East Coast fever alone, caused by *T. parva*, exceed USD 500 million annually across East and Southern Africa, particularly in high-risk areas near protected ecosystems [28,29]. Broader estimates suggest that TBDs cost African livestock systems over $18.7 billion annually, particularly impacting subsistence farmers who depend on livestock for survival [30,31]. Similar concerns arise for *Babesia*, *Anaplasma,* and *Brucella* infections, which compromise reproductive health and productivity in cattle (*Bos taurus* Linnaeus, 1758) and small ruminants [32]. Moreover, wildlife species such as African buffalo (*Syncerus caffer* Sparrman, 1779), impala (*Aepyceros melampus* Lichtenstein, 1812), and warthogs (*Phacochoerus africanus* Gmelin, 1788) can act as asymptomatic reservoirs of these pathogens, sustaining their transmission cycles and complicating control efforts [33,34]. Despite the high burden and risk, information on the prevalence and distribution of ticks and TBDs at wildlife–livestock interfaces remain fragmented and regionally concentrated. Many African countries lack coordinated surveillance and diagnostic infrastructure, leading to data scarcity in vast ecological zones.

The complex dynamics of TBDs are further shaped by environmental, biological, and anthropogenic factors [35]. Tick burden and pathogen prevalence vary across ecological gradients, influenced by seasonality, vector abundance, host density, acaricide resistance, and community-level management practices [17,28]. Tick and pathogen ecology is further affected by climate change, land use, and host availability, with studies showing that tick populations respond to rainfall patterns and livestock presence, which foster tick proliferation [36,37]. Managing tick-borne diseases (TBDs) at the wildlife–livestock interface in Africa is complex due to the ecological interactions among wildlife, livestock, and ticks. This review highlights key tick genera, associated pathogens, host species, and regional prevalence patterns, revealing major surveillance gaps. It emphasizes the need for evidence based, One Health strategies to improve diagnostics, control measures, and policy development for effective TBD management.

## 2. Materials and Methods

### 2.1. Study Area

**Figure 1:**
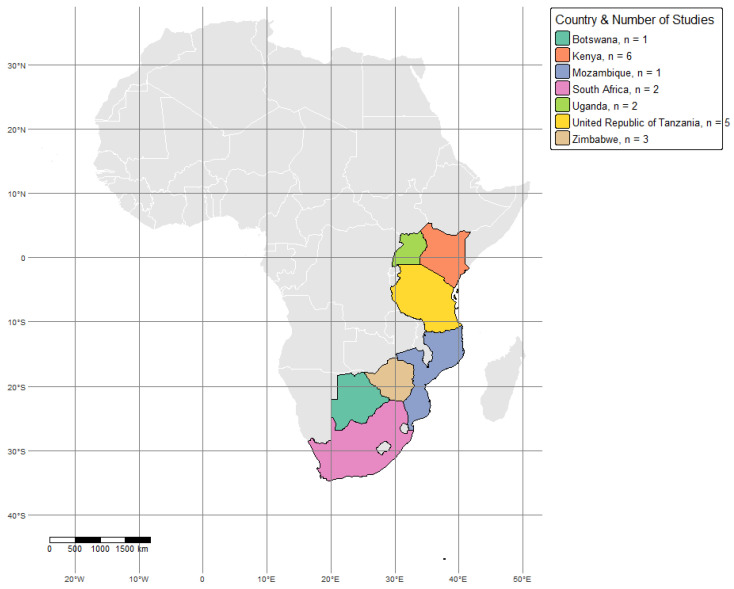
Map of study sites across Africa showing the wildlife–livestock interface locations. Each country is represented with a unique color. The map was generated using R software (version 4.4.1) based on geographic coordinates extracted from the included studies.

As shown in Figure 1, research on the distribution and prevalence of ticks and TBPs in Africa has focused on various wildlife–livestock interface regions, with studies covering multiple ecosystems and conservation areas. This review aimed to assess the distribution and prevalence of tick species and associated pathogens across Africa. However, the final pool of eligible studies retrieved systematically was geographically limited to East, Southern, and a few Central African countries. This reflects the uneven availability of research data across the continent rather than intentional geographic restriction. These regions serve as critical points for pathogen spillover due to close interactions between domestic animals and wildlife. Similarly, in Northern Tanzania, investigations on *T. parva* in asymptomatic cattle near wildlife corridors have highlighted the role of Cape buffaloes (*Syncerus caffer*) as reservoirs for the parasite [38].

Kenya has been a focal point for numerous studies, particularly in the Maasai Mara, Laikipia, and Amboseli ecosystems. Research in Maasai Mara and Laikipia has involved molecular epidemiological assessments of *Rickettsia* and *Coxiella burnetiid* in ticks and wildlife, using PCR and serological methods [33]. In Amboseli, Q fever prevalence and associated risk factors were studied in Impalas (*Aepyceros melampus* Lichtenstein, 1812), sheep (*Ovis aries* Linnaeus, 1758), and goats (*Capra hircus* Linnaeus, 1758), underscoring the potential zoonotic transmission at the interface [39]. In Botswana, studies in the northern region have examined the seroprevalence of *Anaplasma* and *Babesia* spp. in cattle populations coexisting with wildlife. A comparative approach assessed TBP prevalence in areas with and without veterinary fences, providing insights into how physical barriers influence disease transmission [13].

South Africa has also been a key research hub, with studies focusing on TBP seroprevalence in cattle near Kruger National Park. These studies assessed zoonotic pathogens such as *C. burnetii* and *Brucella* spp., utilizing serological methods like ELISA and virus neutralization tests [22]. Across Zimbabwe, wildlife–livestock interface areas in the Great Limpopo Transfrontier Conservation Area have been surveyed for diseases like brucellosis and chlamydiosis, revealing important insights into cross-species disease transmission [22,40].

### 2.2. Search Strategy

This systematic review was designed following the Preferred Reporting Items for Systematic Reviews and Meta-Analyses (PRISMA) guidelines developed Haddaway [41] to assess the prevalence, distribution, and risk factors associated with tick-borne pathogens at the African wildlife–livestock interface. The research question was framed using the PCC (Population, Concept, and Context) framework, with the population being ticks affecting wildlife and livestock in Africa, the concept focusing on distribution, prevalence, and tick-borne pathogens, and the context being the wildlife–livestock interface in African ecosystems. The primary search question guiding this review was: *What is the distribution and prevalence of ticks and tick-borne pathogens at the wildlife–livestock interface in Africa?*

A comprehensive search strategy was developed to retrieve relevant studies from multiple databases, including PubMed/MEDLINE, Web of Science, Scopus, Embase, CABI Global Health, Google Scholar, Dimensions AI, Lens, Core, and Science Direct. The search incorporated controlled vocabulary and free-text keywords related to ticks, tick-borne diseases, wildlife, livestock, and pathogen transmission. Boolean operators (AND, OR) were used to refine search results and optimize retrieval. The search was limited to the last ten years and included only English-language publications to ensure relevance and accessibility. The search strategy involved using different query formulations in each database to maximize retrieval, as shown in Table 1.

### 2.3. Screening Process

The study selection process was conducted in two phases: title and abstract screening, followed by full-text screening. Initially, two independent reviewers screened titles and abstracts to exclude irrelevant studies. Subsequently, full-text reviews were conducted based on predefined inclusion and exclusion criteria. Inclusion criteria encompassed studies reporting the prevalence of tick species and tick-borne pathogens at the wildlife–livestock interface, those providing molecular, serological, or morphological identification of pathogens, and studies evaluating risk factors for disease transmission. Exclusion criteria comprised studies focusing solely on human tick-borne diseases, reviews, conference abstracts, case reports without primary data, and studies with incomplete methodologies.

Duplicate records were identified and removed using various methods, including general duplicate searches, title-based searches, and Levenshtein distance-based searches. Unique references were extracted for the final review. Any disagreements during the screening process were resolved through consensus or consultation with a third reviewer.

### 2.4. Data Extraction and Quality Assessment

Data extraction was performed using a standardized form capturing essential study characteristics such as author, year, location, tick species identified, detected tick-borne pathogens, prevalence rates across host species, host species involved, habitat type, diagnostic methods used, identified risk factors, study design, and sample size. To assess the quality of included studies, we conducted an adapted risk of bias assessment, evaluating selection bias (randomness of sampling, study representativeness), reporting bias (adequacy of diagnostic methods and prevalence reporting), methodology quality (study design and statistical robustness), and overall risk of bias, which was categorized as low, moderate, or high.

### 2.5. Study Inclusion and PRISMA Flow Diagram

**Figure 2:**
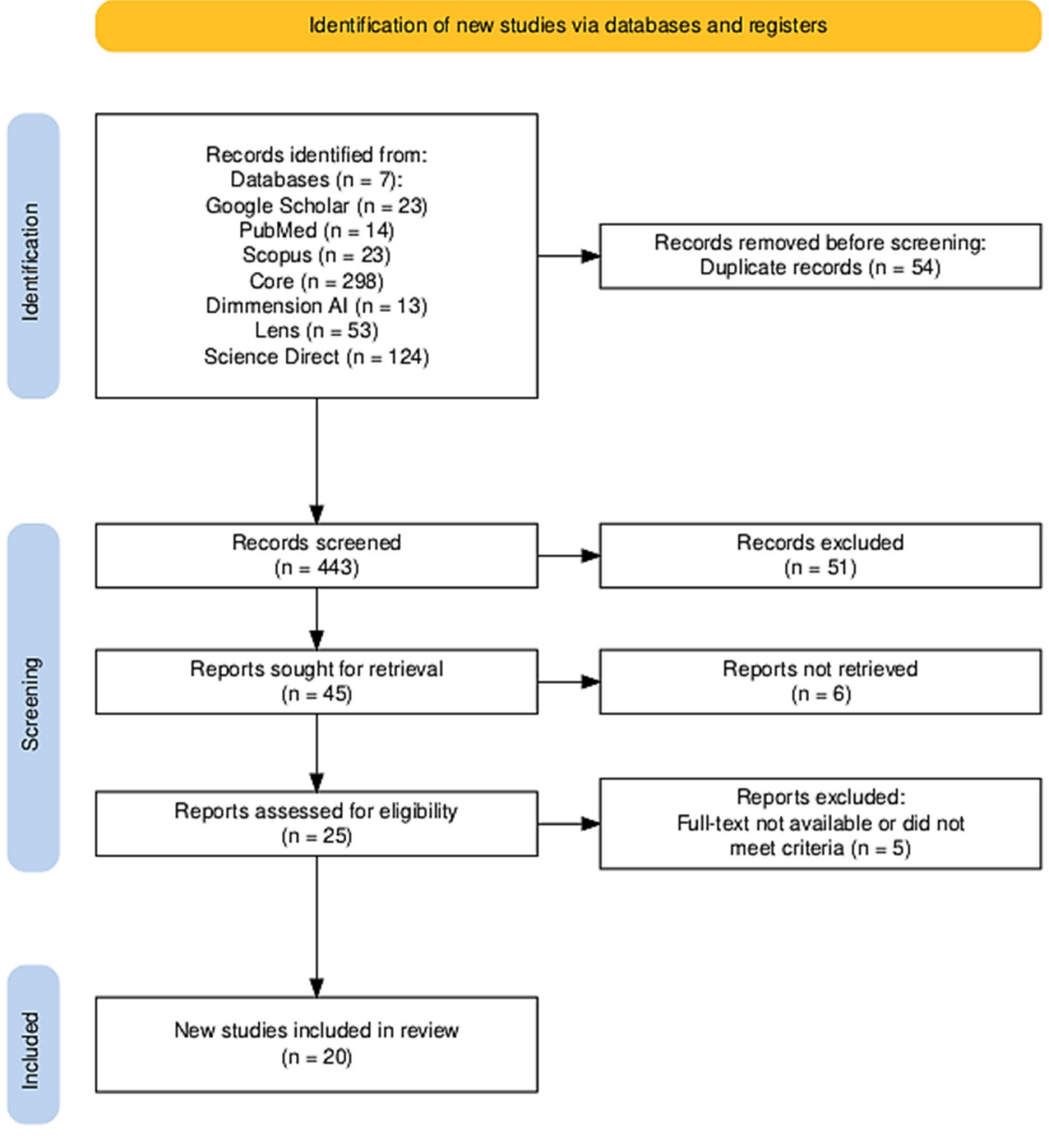
PRISMA Flow Diagram. A flowchart depicting the study selection process for the systematic review. It illustrates the number of records identified from various databases, the number of duplicate records removed, the screening process, the eligibility assessment, and the final number of studies included in the review.

This review was performed in accordance with the PRISMA (Preferred Reporting Items for Systematic Reviews and Meta-Analyses) guidelines. As shown in Figure 2, 548 records were identified across multiple databases. A detailed PRISMA 2020 checklist of reporting items is provided in Appendix A (Table A1) to enhance the methodological transparency of this review. After 54 duplicates were removed, 443 studies remained for title and abstract screening. Following this phase, 25 full-text articles were assessed for eligibility, of which five were excluded due to lack of full-text access or failure to meet inclusion criteria. Consequently, 20 studies, as shown in Table 2, were included in the final systematic review.

This systematic review’s protocol was not registered in any database.

Studies conducted across Africa, as presented in Table 2, demonstrate a wide range of tick-borne and zoonotic pathogens present at the wildlife–livestock interface. In Uganda and Tanzania, *Theileria* species including *T. parva*, *T. velifera*, and *T. mutans* were frequently detected in cattle and small ruminants, alongside other protozoans such as *Babesia bigemina* and *Trypanosoma brucei*. In Zimbabwe and South Africa, livestock populations showed high seroprevalence of *Brucella* spp., *Chlamydia abortus*, and *Coxiella burnetii*, particularly in porous interface areas. In Botswana, a notably high prevalence of *Anaplasma spp.* (up to 90%) was recorded in cattle. Kenyan studies revealed a diverse range of pathogens in cattle, sheep, goats, and wildlife, including *Anaplasma*, *Theileria*, *Babesia*, *Rickettsia*, and *Coxiella burnetii*, with several species also detected in ticks. In Mozambique, African swine fever virus (ASFV) was identified and genotyped in soft ticks collected from warthog burrows and pig shelters. Some studies from Tanzania and Kenya focused primarily on tick species composition or viral metagenomics rather than direct pathogen prevalence in animal hosts.

## 3. Results

**Figure 3:**
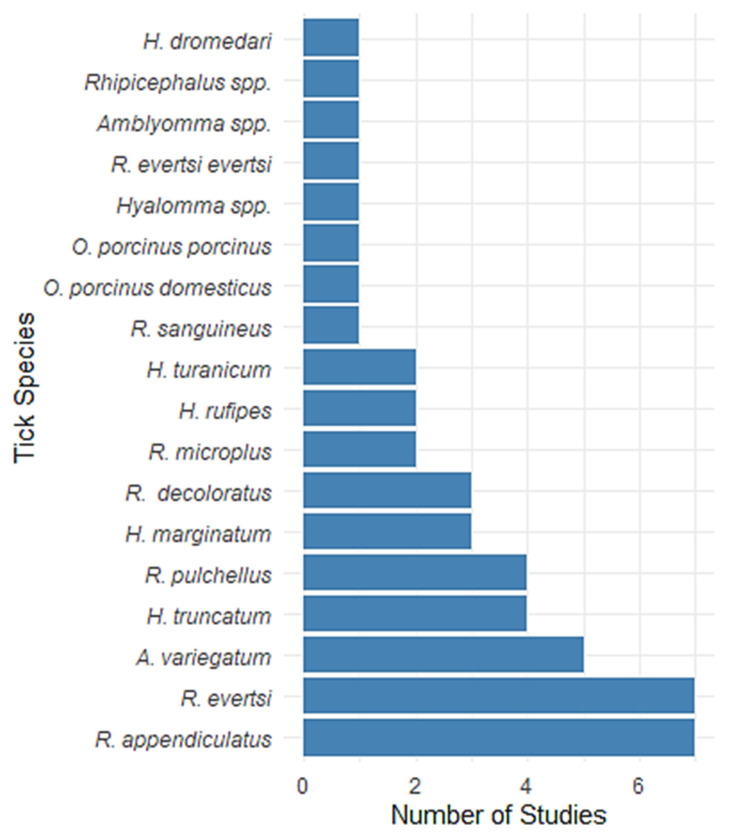
Frequency of tick species reported in studies *A bar chart illustrating the number of studies that reported various tick species in the systematic review*.

Figure 3 presents the number of studies reporting various tick species at the African wildlife–livestock interface. *Rhipicephalus appendiculatus* and *Rhipicephalus microplus* (Canestrini, 1888) were the most frequently reported species, each documented in seven studies. *Amblyomma variegatum* was reported in six studies, followed by *Hyalomma truncatum* Koch, 1844 and *R. pulchellus*, each in four studies. Tick species such as *Hyalomma marginatum* Koch, 1844, *Rhipicephalus decoloratus* (Koch, 1844), and *R. microplus* were reported in three studies each. *Hyalomma rufipes* Koch, 1844, *Hyalomma turanicum* Pomerantzev, 1946, and *Rhipicephalus sanguineus* (Latreille, 1806) appeared in two studies. In contrast, species like *Ornithodoros porcinus domesticus*, *O. porcinus porcinus*, *Hyalomma* spp., *Rhipicephalus evertsi evertsi*, *Amblyomma* spp., *Rhipicephalus* spp., and *Hyalomma dromedari* were reported in only one study each, indicating limited documentation. These frequencies reflect both the distribution of tick species and the focus of current research, with some species potentially underrepresented due to geographic or sampling constraints.

**Figure 4:**
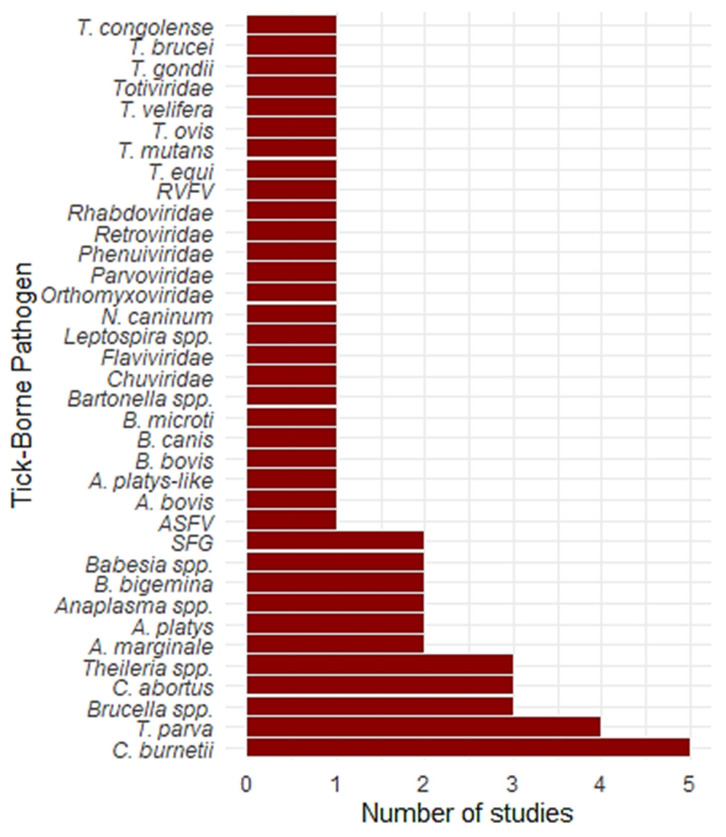
Frequency of tick-borne pathogens reported in studies. *A bar chart illustrating the number of studies that reported various tick-borne pathogens in the systematic review*.

Figure 4 presents the number of studies that reported various tick-borne pathogens at the African wildlife–livestock interface. *Coxiella burnetii* was the most frequently reported pathogen, appearing in five studies. This was followed by *Theileria parva* and *Brucella* spp., each reported in four studies. *Chlamydia abortus* and *Theileria* spp. were identified in three studies. *Anaplasma marginale*, *Anaplasma platys*, *Anaplasma* spp., *Babesia bigemina*, and *Babesia* spp. were each reported in two studies.

Several other pathogens were reported in only one study. These included *African swine fever virus (ASFV)*, spotted fever group (SFG) *Rickettsia*, *Anaplasma bovis*, *A. platys-like*, *Babesia bovis*, *Babesia canis*, *Babesia microti*, *Bartonella* spp., and *Leptospira* spp. In addition, several viral families were reported only once. These included *Chuviridae*, *Flaviviridae*, *Orthomyxoviridae*, *Parvoviridae*, *Phenuiviridae*, *Retroviridae*, and *Rhabdoviridae*. The protozoan pathogens *Neospora caninum*, *Toxoplasma gondii*, *Theileria equi*, *Theileria mutans*, *Theileria ovis*, *Theileria velifera*, *Trypanosoma brucei*, and *Trypanosoma congolense* were each reported in only one study. While the primary vectors for many *Trypanosoma* species are blood-feeding invertebrates like tsetse flies, emerging research suggests that ticks may also act as vectors for certain *Trypanosoma* species, and the presence of these parasites in ticks warrants further investigation into their potential role in transmission [52,53,54].

**Figure 5:**
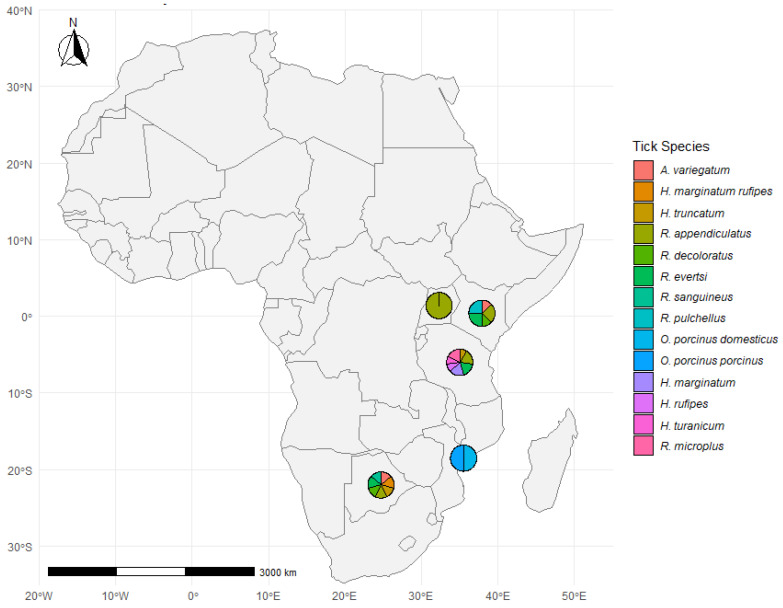
Distribution of tick species across study sites in Africa. *A geographic representation of tick species distribution at various study sites across Africa. The pie charts at each location indicate the proportion of different tick species identified in the respective regions. The color legend represents the tick species.* The map and visualizations were created using R software (version 4.4.1), with tick species data summarized per site.

Figure 5 displays the geographic distribution of tick species reported in 20 studies conducted across seven African countries. The majority of data points are concentrated in East and Southern Africa, particularly in Uganda, Kenya, Tanzania, Zimbabwe, Mozambique, Botswana, and South Africa. Each pie chart represents the diversity of tick species recorded in specific study locations, showing variation in species composition across regions.

Across all sites, the most commonly recorded tick genera were *Rhipicephalus*, *Amblyomma*, *Hyalomma*, and *Ornithodoros*. Among the species, *Rhipicephalus appendiculatus* and *Amblyomma variegatum* were the most frequently reported, consistent with their known wide distribution and role in pathogen transmission. *R. appendiculatus* appeared in seven studies, while *A. variegatum* was reported in six studies. Other species such as *R. evertsi*, *Hyalomma truncatum*, *R. pulchellus*, and *H. marginatum* were also recorded in multiple regions, illustrating the broad ecological range of these vectors. In contrast, *Ornithodoros porcinus porcinus* and *O. porcinus domesticus* were reported in a single location in Mozambique, indicating more localized detection.

**Figure 6:**
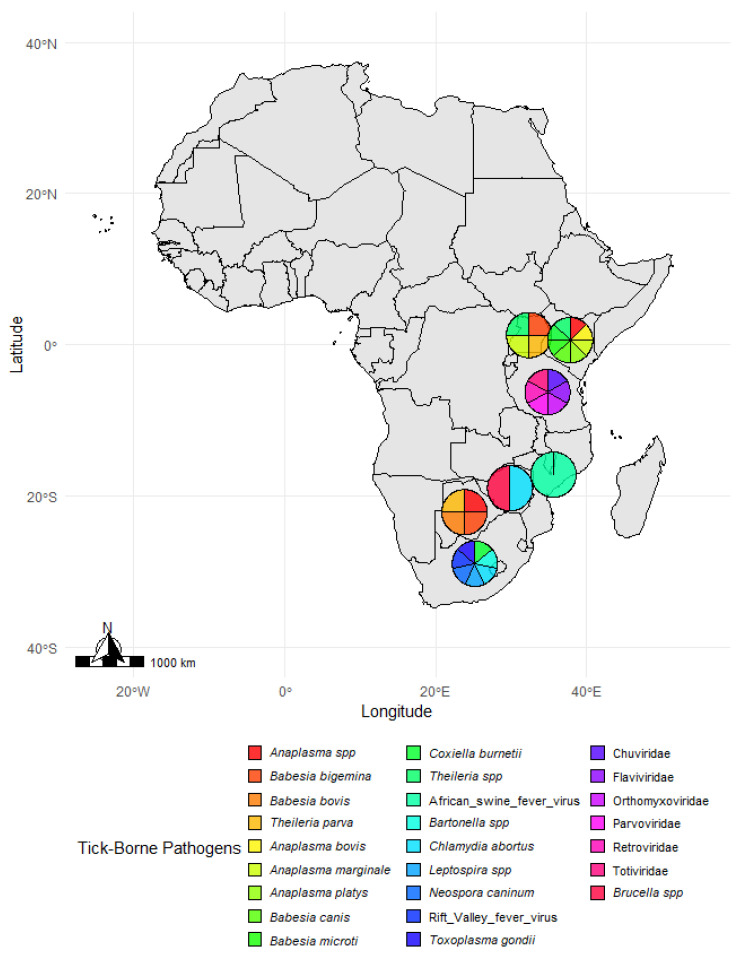
Tick species distribution across study sites in Africa. *A map illustrates the distribution of tick species at various study sites across Africa. Pie charts at each location represent the proportion of different tick species identified in the respective regions. The color legend at the bottom corresponds to specific tick species, allowing for a visual comparison of their geographic distribution.* The map was generated in R (version 4.4.1) using species data and spatial coordinates from the reviewed literature.

Tick-borne pathogens vary widely across African regions, as shown in Figure 6, with notable diversity in Uganda and South Africa. This suggests that local biodiversity and interactions between wildlife and livestock drive the transmission of these pathogens. High pathogen diversity in Uganda’s Queen Elizabeth National Park and South Africa’s Kruger National Park, including zoonotic agents like *C. burnetii* and Rift Valley fever virus, emphasizes the need for ongoing surveillance and targeted interventions. Botswana and Kenya face significant livestock health risks from *Theileria* and *Anaplasma* species, calling for improved tick control and vaccination strategies. Mozambique’s African swine fever virus prevalence highlights the interconnected nature of tick-borne and vector-borne diseases, emphasizing the need for integrated research and monitoring efforts.

**Figure 7:**
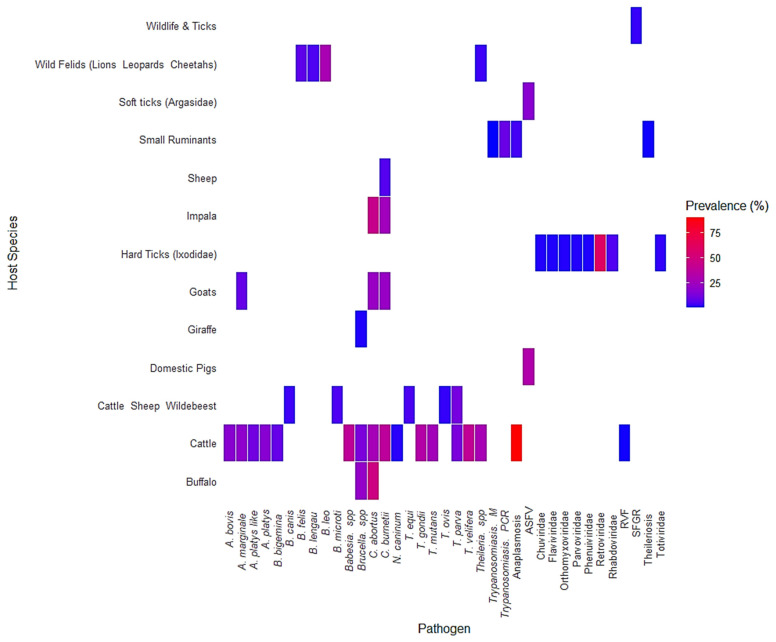
Prevalence of tick-borne pathogens across host species. *A heatmap illustrating the prevalence of various tick-borne pathogens in different host species. The color gradient represents prevalence percentages, with red indicating high prevalence and blue indicating low prevalence*.

Figure 7 presents a heatmap showing the prevalence of various tick-borne pathogens across different host species. The color scale ranges from blue, indicating low prevalence, to red, indicating high prevalence. Cattle (*Bos taurus* Linnaeus, 1758) were the most frequently sampled host species and exhibited the broadest range of pathogens. These included *Anaplasma marginale*, *A. platys*, *A. bovis*, *Babesia bigemina*, *Brucella* spp., *Coxiella burnetii*, and *Theileria parva*, with several pathogens reaching high prevalence values above 50%, as shown in red. Buffalo (*Syncerus caffer*) were also found to harbor multiple pathogens, including *Theileria* spp., *Brucella* spp., and *Babesia* spp., although prevalence levels were generally lower compared to cattle. Impala (*Aepyceros melampus*) had detectable levels of *Theileria parva*, *Babesia* spp., and *Coxiella burnetii*, with some infections exceeding 25% prevalence. Goats (*Capra hircus*) and sheep (*Ovis aries*) showed limited but notable occurrences of *Brucella* spp. and *Coxiella burnetii*. In domestic pigs (*Sus scrofa domesticus* Erxleben, 1777), *Brucella* spp. was detected at moderate prevalence. Tick vectors, both hard ticks (*Ixodidae*) and soft ticks (*Argasidae*), were linked to several pathogens, including *Anaplasma* spp., *Babesia* spp., *Theileria* spp., and viruses from the *Flaviviridae* and *Rhabdoviridae* families. This reinforces their established role as reservoirs and vectors of disease at the wildlife–livestock interface. Wild felids such as lions (*Panthera leo* Linnaeus, 1758), leopards (*Panthera pardus* Linnaeus, 1758), and cheetahs (*Acinonyx jubatus* Schreber, 1775) were associated primarily with *Babesia* spp., although the data on prevalence were more limited. Other wildlife, including giraffes (*Giraffa camelopardalis* Linnaeus, 1758), warthogs (*Phacochoerus africanus* Gmelin, 1788), and bushpigs (*Potamochoerus larvatus* F. Cuvier, 1822), were infrequently sampled and showed low detection of pathogens such as *Coxiella burnetii* and ASFV (*African swine fever virus*).

**Figure 8:**
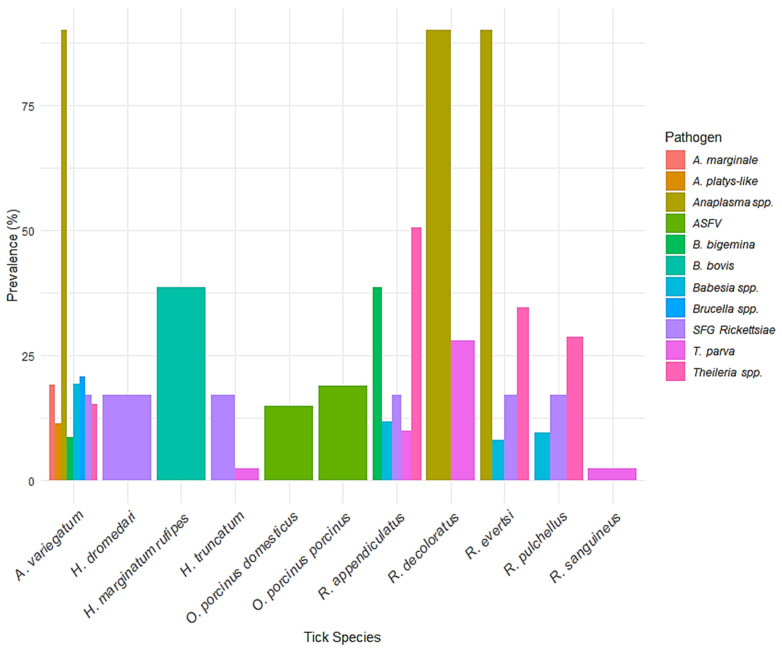
Prevalence of tick-borne pathogens across tick species. *A bar chart illustrating the prevalence of various tick-borne pathogens in different tick species. The color legend indicates the specific pathogens detected*.

Figure 8 illustrates the prevalence of tick-borne pathogens across different tick species. *Rhipicephalus decoloratus* exhibited the highest recorded prevalence, with *Anaplasma* spp. detected in 90% of samples. This was followed closely by *R. evertsi* and *Amblyomma variegatum*, both also showing 90% prevalence for *Anaplasma* spp., indicating their potential significance as vectors of this bacterial pathogen. *A. variegatum* further displayed notable levels of *Brucella* spp. (20.7%), *A. marginale* (19.2%), *Babesia* spp. (19.4%), and *A. platys-like* (11.5%). In contrast, *R. appendiculatus* was associated with a broader range of pathogens, including *Theileria* spp. (50.5%), *B. bigemina* (38.6%), *Babesia* spp. (11.8%), and *SFG Rickettsiae* (17.2%). Soft ticks such as *Ornithodoros porcinus porcinus* and *O. porcinus domesticus* were exclusively associated with African swine fever virus (*ASFV*), at 19% and 15% prevalence, respectively. Among the *Hyalomma* species, *H. marginatum rufipes* showed a 38.6% prevalence of *B. bovis*, while *H. truncatum* had relatively lower detections, with *T. parva* (2.4%) and *SFG Rickettsiae* (17.2%). *R. pulchellus* and *R. sanguineus* were mainly associated with *T. parva* and *SFG Rickettsiae*, although at lower prevalence levels.

**Figure 9:**
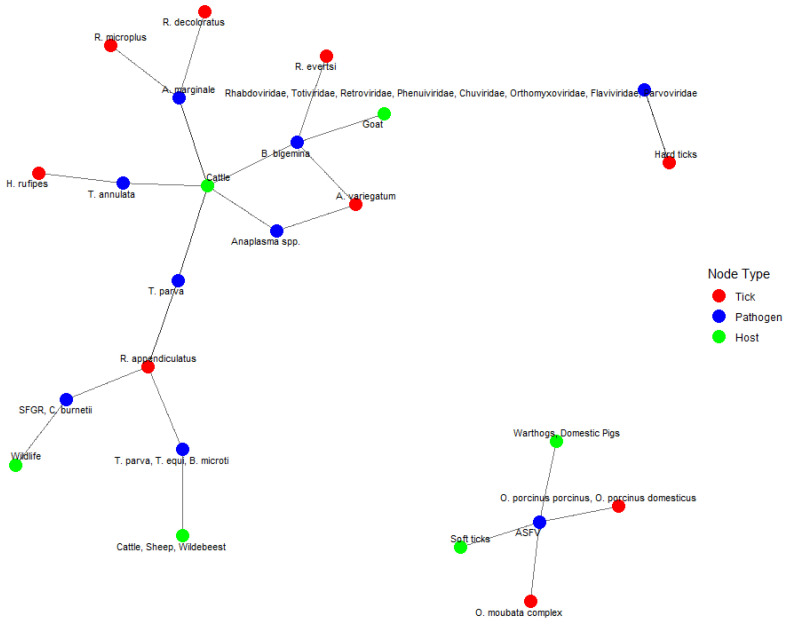
Network graph of tick–pathogen–host interactions. *A network diagram illustrating the relationships between tick species (red nodes), pathogens (blue nodes), and host species (green nodes). The connections represent known associations between ticks, the pathogens they transmit, and the host species they infect*.

Figure 9 presents a network graph illustrating the interactions among 10 tick species, 18 pathogens, and 14 host species identified in the dataset. The network captures a total of 13 tick–pathogen interactions, 22 pathogen–host interactions, and 15 tick–host interactions, indicating multiple points of overlap and potential transmission routes within the system. *Cattle* (green node) was the most frequently connected host species, with direct links to multiple tick species, including *Rhipicephalus appendiculatus*, *R. microplus*, *R. decoloratus*, and *Hyalomma rufipes*, as well as pathogens such as *Anaplasma marginale*, *Babesia bigemina*, *Theileria parva*, and *Coxiella burnetii*. This reflects the intensive surveillance of cattle and their central role in the epidemiology of tick-borne diseases. Among tick vectors, *R. appendiculatus* (red node) had the highest number of interactions. It was connected to at least five pathogens, including *T. parva*, *T. equi*, *Babesia microti*, *C. burnetii*, and *SFG Rickettsia*, and was linked to both domestic hosts (cattle, sheep, wildebeest) and wildlife. This high degree of connectivity indicates its significant role as a bridge vector between wildlife and livestock. The most frequently reported pathogen was *Anaplasma marginale* (blue node), which was associated with at least three tick species (*R. microplus*, *R. decoloratus*, and *A. variegatum*) and was detected in cattle. Similarly, *B. bigemina* showed connections with multiple ticks and hosts, underscoring its broad transmission potential. The network also identifies distinct clusters. For example, the *Ornithodoros porcinus porcinus* and *O. porcinus domesticus* ticks were uniquely associated with *ASFV* and showed exclusive interactions with warthogs and domestic pigs. Another cluster grouped soft ticks and *Ornithodoros moubata* (Murray, 1877) *complex* with *ASFV*, forming a separate interaction group with wildlife and pigs. In addition, the tick *R. evertsi* was linked to multiple viral families, including Rhabdoviridae, Retroviridae, Totiviridae, Phenuiviridae, Chuviridae, Orthomyxoviridae, Flavivirus, and Parvovirus, with goat as a reported host. These associations suggest emerging areas of pathogen diversity requiring further investigation

**Figure 10:**
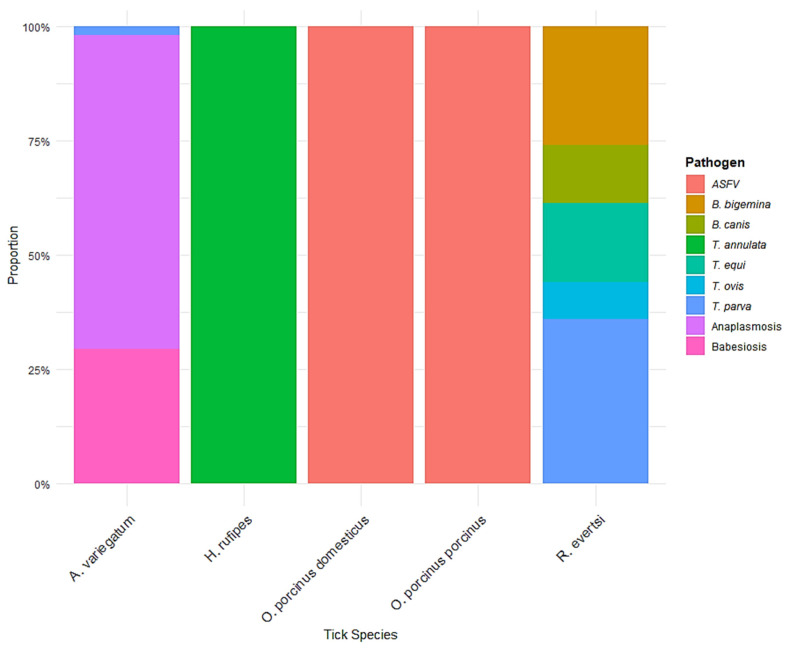
Proportional distribution of pathogens across tick species. *A stacked bar chart illustrating the proportion of different pathogens detected in various tick species. The color legend represents the specific pathogens identified*.

Figure 10 displays the proportional distribution of pathogens associated with five tick species based on the dataset. These species include *Amblyomma variegatum*, *Hyalomma rufipes*, *Ornithodoros porcinus domesticus*, *O. porcinus porcinus*, and *Rhipicephalus evertsi*. *Amblyomma variegatum* exhibited the highest pathogen diversity among the tick species analyzed. Approximately 65% of its pathogen associations were with *Anaplasmosis*, followed by *Babesia canis* (around 30%), and *Theileria parva* (around 5%). *Hyalomma rufipes* was almost exclusively associated with *Babesiosis*, accounting for 100% of its pathogen linkage in this dataset. *Ornithodoros porcinus domesticus* and *O. porcinus porcinus* showed a complete (100%) association with *African Swine Fever Virus (ASFV)*, with no evidence of co-infection or association with other pathogens within the reviewed studies. This suggests a specific and possibly exclusive relationship between these soft ticks and ASFV in the sampled regions. *Rhipicephalus evertsi* showed the most varied pathogen profile. It was associated with *Theileria parva* (approximately 35%), *Theileria equi* (20%), *Theileria ovis* (15%), *Babesia bigemina* (10%), *Babesia canis* (10%), and *Babesiosis* (10%).

Diagnostic Methods for Tick and Tick-Borne Pathogen Detection at the Wildlife–Livestock Interface.

Various diagnostic techniques have been employed to investigate ticks and tick-borne pathogens (TBPs) distribution and prevalence at the wildlife–livestock interface across Africa. Traditional morphological identification remains fundamental for characterizing tick species, often using taxonomic keys for genus and species-level classification. However, molecular tools have significantly enhanced precision in identifying ticks and associated pathogens. Molecular techniques such as polymerase chain reaction (PCR) and high-resolution melting (HRM) analysis have been extensively used to detect bacterial and protozoan TBPs. For example, studies in Maasai Mara and Laikipia, Kenya, utilized PCR and HRM analyses to detect *Rickettsia* spp. and *C. burnetii*, the causative agent of Q fever [33]. Additionally, reverse line blot hybridization (RLB) has been employed for the molecular characterization of *Theileria* and *Babesia* spp. in Kenya [50], while real-time PCR targeting the IS1111a gene has been instrumental in detecting *C. burnetii* in ticks and wildlife [33].

Metagenomic sequencing techniques have also revolutionized the study of TBPs, particularly in tick virology. In Mikumi National Park, Tanzania, viral metagenomic approaches, including Illumina high-throughput sequencing, were used to identify a range of viral families within tick populations, demonstrating the broad diversity of tick-associated viruses [43]. Serological methods remain essential for detecting exposure to TBPs in livestock and wildlife populations. Enzyme-linked immunosorbent assays (ELISA) and competitive inhibition ELISA (cELISA) have been used extensively to determine the seroprevalence of *Anaplasma* spp., *T. parva*, and *Babesia* spp. in cattle at wildlife–livestock interface regions, such as Northern Botswana and South Africa’s Kruger National Park [13,22]. The Indirect Fluorescence Antibody Test (IFAT) has also been applied to detect antibodies against protozoan TBPs in African livestock [13].

Phylogenetic reconstruction and sequence analysis have been employed for genetic diversity analysis to study TBP variation across regions. For example, in Northern Tanzania, PCR-based analysis of *T. parva* p67 gene polymorphisms was used to assess genetic diversity in asymptomatic cattle, revealing the presence of buffalo-derived strains [38].

**Figure 11:**
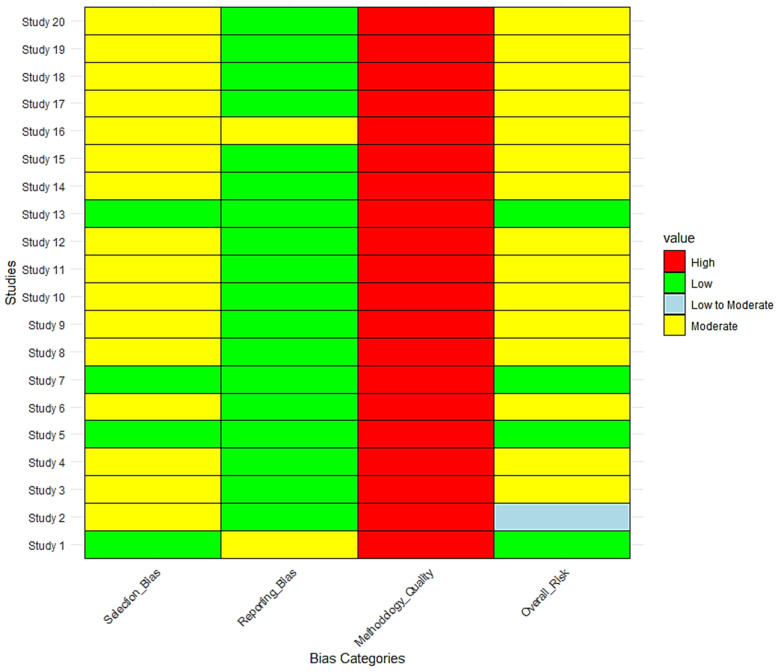
Risk of bias assessment across studies at the wildlife–livestock interface.

As shown in Figure 11, The risk of bias assessment across 20 studies on tick-borne diseases at the wildlife–livestock interface revealed that while methodological quality was consistently high (100% of studies, n = 20), selection bias and sampling limitations contributed to moderate overall bias in 65% of studies (n = 13). Only 20% of studies (n = 4; Study 1, Study 5, Study 7, and Study 13) had a low overall risk of bias, indicating strong random sampling, robust molecular diagnostics, and statistical adjustments. In contrast, one study (Study 2, 5%) had a low-to-moderate risk, while the remaining 75% (n = 15) had a moderate overall risk due to non-randomized site selection, limited geographic scope, and potential underrepresentation of wildlife hosts.

Regarding selection bias, only 20% (n = 4; Study 1, Study 5, Study 7, and Study 13) had low risk, while 80% (n = 16) exhibited moderate selection bias, primarily due to cluster-based or convenient sampling (Study 2, Study 3, Study 6, Study 9). Reporting bias was low in 90% of studies (n = 18), with most studies using validated diagnostic methods (PCR, ELISA, sequencing). However, two studies (Study 1 and Study 16, 10%) had moderate reporting bias, primarily due to limited discussion on the potential underreporting of co-infections and restricted focus on livestock hosts without vector sampling.

Although the methodological quality was consistently high (100% of studies, n = 20), limitations arose in sampling constraints and a lack of confirmatory molecular testing for serology-based studies (40%, n = 8; Study 4, Study 8, Study 10, Study 11).

This heatmap visualizes the risk of bias assessment for 20 studies investigating tick-borne diseases and pathogen prevalence at the wildlife–livestock interface. The four bias categories assessed include Selection Bias, Reporting Bias, Methodology Quality, and Overall Risk. Color coding represents different risk levels: Green (Low Risk), Yellow (Moderate Risk), Light Blue (Low to Moderate Risk), and Red (High Risk).

## 4. Discussion

### 4.1. Prevalence and Distribution of Tick Species

The review identified *R. appendiculatus*, *R. evertsi*, *A. variegatum*, and *Hyalomma truncatum* as the most frequently reported tick species. These species have a broad host range and are widely distributed across ecological zones [55]. *Rhipicephalus appendiculatus* thrives in moist, cooler environments, typically at higher altitudes with moderate temperatures ranging between 15–26 °C. It prefers areas with good vegetation cover and high humidity, often in regions receiving moderate rainfall but less prevalent in hot, dry areas [56]. In Uganda, *R. appendiculatus* was predominantly found in Queen Elizabeth National Park, particularly in cattle and buffalo populations, with a prevalence of 50.5%. In contrast, *R. evertsi* adapts to moderately warm climates with lower humidity and is commonly found in subtropical shrublands. It tolerates a broader range of temperatures and rainfall compared to *R. appendiculatus* [57,58]. Studies in Botswana indicate *R. evertsi* is frequently reported in wildlife conservation areas, sustaining its population through host interactions with domestic and wild animals. *Amblyomma variegatum* favors warmer, humid environments and is frequently found in tropical grasslands with dense vegetation, providing ample access to hosts [59,60]. In Uganda, *A. variegatum* was commonly detected in cattle at Queen Elizabeth National Park, with an estimated prevalence of 19.2%.

Meanwhile, *H. truncatum* is well-suited to arid and semi-arid regions with sparse vegetation and low rainfall. It thrives in high-temperature areas and is often associated with livestock housing [61,62]. The study in Botswana has shown that tick species’ distribution patterns are influenced by wildlife migration, particularly in transboundary conservation areas where the free movement of hosts sustains tick populations year-round [13]. *Rhipicephalus microplus* is increasingly spreading across East Africa, driven by climate change and livestock trade, indicating a significant ecological shift in tick distribution [38]. In addition, some studies suggest that increased human settlement and deforestation have disrupted tick–host relationships, forcing ticks to seek alternative hosts and facilitating their spread into new ecosystems [49].

The high prevalence of *R. appendiculatus* in cattle and buffalo is particularly concerning, as this tick is a primary vector of *T. parva*, the causative agent of East Coast fever (ECF) [63]. The presence of *R. appendiculatus* in buffalo and cattle is supported by Maboko [64], who reported that buffaloes serve as the parasite’s original host for *T. parva*, while cattle have become its more recent host. Its presence in Queen Elizabeth National Park highlights its role in maintaining the transmission cycle of *T. parva* between buffalo and cattle, a significant concern for disease management in Uganda. Moreover, *R. evertsi* transmits *Babesia caballi* and *T. equi (equine piroplasmosis)*, *Theileria separata (ovine theileriosis)*, and *A. marginale (anaplasmosis in cattle)* [65], *A. variegatum* spreads heartwater (*Ehrlichia ruminantium*), and African tick-bite fever (*R. africae*) [66] and *H. truncatum* is a key vector of the Crimean-Congo hemorrhagic fever (CCHF) virus [67]. The spread of invasive tick species like *H. rufipes* into new areas, facilitated by livestock trade and movement, raises concerns about emerging tick-borne diseases, including Crimean-Congo hemorrhagic fever [33,50].

Regional variation in tick species distribution was evident across the reviewed studies. Countries such as Kenya, Tanzania, South Africa, Botswana, Uganda, and Zimbabwe were identified as key hotspots for tick infestations. In Uganda, *R. appendiculatus*, *Rhipicephalus decoloratus*, and *A. variegatum* were frequently reported in wildlife conservation areas, including Queen Elizabeth National Park and Budongo Conservation Forest. In Kenya, the predominant species reported included *R. appendiculatus*, *R. evertsi*, *R. pulchellus*, and *A. variegatum*, with some regions also recording *H. truncatum* and *H. dromedari*. Tanzania exhibited a high diversity of tick species, with *R. microplus*, *R. evertsi*, *H. rufipes*, *H. truncatum*, *Hyalomma marginatum*, and *Hyalomma turanicum* being frequently reported. Botswana recorded a mixture of *Rhipicephalus*, *Amblyomma*, and *Hyalomma* species. At the same time, Uganda had significant records of *R. appendiculatus*, *R. decoloratus*, and *A. variegatum*, particularly in wildlife conservation areas such as Queen Elizabeth National Park and Budongo Conservation Forest. Interestingly, studies from Uganda suggest that tick populations in wildlife-dominated ecosystems may be underreported due to limited surveillance efforts, necessitating targeted epidemiological studies in these regions [33]. Despite the significance of tick-borne diseases, surveillance in West and Central Africa remains sparse. This lack of data prevents a comprehensive understanding of tick diversity and emerging pathogens, highlighting the need for increased monitoring in these regions [50].

Remarkably, *R. microplus* in Tanzania suggests a possible expansion of this invasive species within East Africa, aligning with previous reports of its spread from southern Africa. The detection of *O. porcinus porcinus* and *O. porcinus domesticus* in Mozambique highlights the role of soft ticks in African swine fever virus (ASFV) epidemiology, emphasizing the need for vector-targeted surveillance and control in wine-producing regions. Tick distribution is influenced by diverse habitats, seasonal variations, and host characteristics such as age and location [4,68,69]. The variations in tick prevalence highlight the importance of targeted control strategies that consider local ecological and climatic conditions. Tick management may require a One Health approach, integrating wildlife, livestock, and environmental health in wildlife-dominated ecosystems such as the Serengeti and Queen Elizabeth National Park. Moreover, the lack of data from Central and West Africa underscores the need for expanded tick surveillance to better understand distribution patterns and emerging risks. Countries such as Kenya, Tanzania, South Africa, Botswana, and Uganda emerged as hotspots for tick infestations. In these environments, the interactions between wild and domesticated animals facilitate complex dynamics of tick ecology, resulting in higher infestation rates [70,71]. The review also highlights a lack of comprehensive surveillance in certain regions, particularly Central and West Africa, which may contribute to gaps in understanding tick distribution. The adaptability of *R. evertsi and A. variegatum* further complicates their management and contributes to their widespread distribution across sub-Saharan Africa [72,73].

### 4.2. Pathogen Prevalence and Host Interactions

Among tick-borne pathogens, *T. parva*, *A. marginale*, *B. bigemina*, *Brucella* spp., and *C. burnetii* were the most frequently reported in the studies reviewed. *T. parva* was predominantly detected in *R. appendiculatus*-infested cattle and buffalo in Queen Elizabeth National Park, reinforcing its role in ECF transmission with a prevalence of 50.5%. Further supporting the findings, it is well-established that *R. appendiculatus* is the key tick vector for *T. parva*, as it is identified as the principal transmitter of the pathogen responsible for East Coast fever (ECF) in cattle [74]. Additionally, studies have shown that the vector plays a crucial role in the transmission dynamics of *T. parva* in endemic areas, contributing to the high morbidity and mortality associated with the disease [75]. *A. marginale* was commonly detected in *R. microplus* and *R. evertsi*, implicating them in bovine anaplasmosis transmission. This finding supports the report by Ruybal [76], which highlights that *A. marginale*, prevalent in tropical and subtropical regions, poses a significant challenge to cattle production in areas with endemic tick infestations, as it causes bovine anaplasmosis. *A. marginale* was frequently identified in *R. microplus* and *R. evertsi* in cattle from Uganda, with an observed prevalence of 27.9%. Co-infections of multiple tick-borne pathogens have been increasingly reported, with studies in Maasai Mara and Serengeti ecosystems identifying simultaneous infections of *T. parva* and *A. marginale*, exacerbating disease severity and complicating treatment outcomes [49].

The review also revealed significant cross-species pathogen transmission, with pathogens detected in multiple host species, including cattle, buffalo, impala, domestic pigs, warthogs, and bushpigs. These findings are supported by Espinaze [77], those who demonstrated that large and medium-sized mammal hosts are highly connected through shared tick species, facilitating cross-infestation and pathogen transmission. The review highlighted the significant role of domestic animals in maintaining network connectivity, reinforcing evidence of pathogens spread across multiple host species. Cattle, in particular, exhibited the highest pathogen burden, with notable occurrences of *Anaplasmosis*, *Brucella* spp., and *Theileria* spp. This may be attributed to their frequent exposure to tick vectors and interactions with domestic and wild animals, especially in grazing systems near wildlife reserves. In contrast, buffalo and impala exhibited lower pathogen prevalence, with only *T. parva* and *Babesia* spp. detected.

Other studies have reported that although asymptomatic buffalo pose a risk to livestock as reservoir hosts. *E. ruminantium*, the causative agent of heartwater, is transmitted by *Amblyomma* three-host ticks [78]. Similarly, wild felids such as lions, leopards, and cheetahs were primarily associated with *B.* infections, suggesting their role as incidental hosts acquiring pathogens from prey species. To mitigate the risk of cross-species transmission of tick-borne pathogens, controlled grazing strategies should be implemented to minimize direct contact between cattle and wildlife, enhance biosecurity measures for domestic livestock, and promote targeted tick control interventions in high-risk areas. Furthermore, advancements in molecular diagnostics, including PCR-based genotyping of *T. parva* variants, enhance our understanding of pathogen diversity and its implications for vaccine development [33]. Understanding the genetic diversity of tick-borne pathogens and their vectors can help design region-specific control measures that integrate environmental, veterinary, and public health perspectives.

Soft ticks (*Ornithodoros* spp.) were highly associated with the African Swine Fever Virus (ASFV), confirming their role in the epidemiology of African swine fever (ASF). Although ASFV is not traditionally classified as a tick-borne disease, its transmission dynamics involving soft ticks highlight the need for integrated vector control measures. Only specific Argasidae species are confirmed vectors of ASFV, with eight taxa identified in laboratory and field studies [79]. The detection of ASFV DNA in 19% of *O. p. porcinus* and 15% of *O. p. domesticus* further supports the role of soft ticks in maintaining the virus within wildlife reservoirs, with three identified genotypes—Genotype II (linked to European and Malagasy strains), Genotype V (previously found in Mozambique and Malawi), and a newly identified Genotype XXIV—demonstrating the genetic diversity and potential for regional ASFV spread. These findings confirm the risk of ASFV spillover from wildlife to domestic pig populations, underscoring the importance of continuous surveillance of the sylvatic cycle. To mitigate the risk of ASF outbreaks, it is crucial to implement targeted vector control strategies, strengthen biosecurity measures in pig farming systems, and enhance surveillance efforts in areas where soft ticks are prevalent. Additionally, understanding the genetic diversity of ASFV strains can aid in designing region-specific control and prevention strategies.

The assessment of bias and methodology in the reviewed studies revealed variations in study design, diagnostic approaches, and geographic coverage, impacting the overall reliability of findings. Selection bias was a common concern, particularly in studies that employed convenience sampling or had limited geographic representation. While some studies, such as Byamukama [16], mitigated selection bias through random sampling, others, including the prevalence study on hemoprotozoan parasites in Uganda, exhibited moderate selection bias due to cluster-based sampling or restricted livestock sampling [23]. Studies focusing on tick diversity, such as the phylogenetic analysis of hard ticks in Tanzania, demonstrated low selection bias by incorporating systematic tick collection from multiple ecological zones, ensuring a comprehensive representation of tick species diversity [38].

Reporting bias was generally low across most studies, primarily due to standardized diagnostic tools, including PCR, ELISA, metagenomics, and serological assays. Studies that reported prevalence estimates alongside detailed methodologies, such as the seroepidemiological study in South Africa and the molecular survey of *Coxiella burnetii* in Kenya, minimized reporting bias [22,45]. However, some studies exhibited moderate reporting bias due to limited discussion on potential co-infections, underreporting of diagnostic limitations, or reliance on microscopy alone for pathogen detection. For instance, the molecular survey of tick species in Kenya documented diverse tick populations but lacked direct vector-host association data. At the same time, studies relying on microscopy, such as the hemoprotozoan parasite study in Uganda (2019), risked underreporting due to the absence of molecular confirmation [23,49].

Methodological quality varied across studies, with those integrating molecular techniques such as PCR, sequencing, and metagenomics ranking higher in reliability. Studies that incorporated phylogenetic analysis, spatial mapping, and statistical adjustments for clustering, such as the diversity of viruses in hard ticks and the molecular survey of *Theileria* and *Anaplasma* in Kenya, demonstrated robust methodological frameworks [44]. However, some studies relied solely on serological assays without confirmatory PCR testing, potentially overestimating pathogen exposure compared to active infections. For example, the seroprevalence study in Botswana used ELISA and IFAT but lacked PCR confirmation, leading to a moderate overall risk of bias [13]. Similarly, studies on *Brucella* and *Chlamydia* seroprevalence in Zimbabwe employed serological methods but lacked molecular diagnostics, raising concerns about potential misclassification of infections [34].

Studies using randomized sampling, molecular diagnostics, and robust statistical methods had the lowest risk of bias [80]. Conversely, studies with convenience sampling, limited confirmatory diagnostics, or restricted geographic scope exhibited moderate risk of bias. Key limitations across studies included insufficient wildlife sampling, with most research focusing on livestock hosts, potentially underestimating the role of wildlife reservoirs in pathogen transmission. Additionally, the cross-sectional design of many studies limited the ability to assess seasonal and long-term trends in tick-borne pathogen prevalence. Furthermore, while many studies confirmed pathogen presence, few investigated vector competence or transmission dynamics, limiting their implications for disease control.

### 4.3. Implications for Disease Control

The findings underline critical aspects of tick and tick-borne disease management. Strengthening epidemiological surveillance is vital for detecting emerging tick-borne pathogens, while future research should prioritize expanding pathogen screening across diverse habitats and host species to address existing knowledge gaps. Given the high prevalence of pathogens in cattle, targeted interventions such as vaccination programs, acaricide application, and rotational grazing should be prioritized in areas with high livestock density.

Detecting zoonotic pathogens (e.g., *Brucella* spp., *C. burnetii*) in livestock and wildlife highlights the need for interdisciplinary collaboration between veterinary, public health, and environmental sectors. Conventional tick control methods, such as acaricide use, may be ineffective in regions where wildlife is a continuous reservoir for ticks. Therefore, integrated tick management strategies should incorporate biological control, habitat modification, and targeted vector control programs to enhance effectiveness.

Future research should explore the influence of climate change, land use alterations, and ecological shifts on tick populations and pathogen transmission. A better understanding of these factors will support the development of predictive models for tick-borne disease outbreaks and inform sustainable, long-term management strategies.

## 5. Conclusions

This review comprehensively summarizes the distribution and prevalence of ticks and tick-borne pathogens at Africa’s wildlife–livestock interface. The most commonly reported tick vectors were *Rhipicephalus appendiculatus*, *R. evertsi*, and *Amblyomma variegatum*, while the leading pathogens included *Theileria parva*, *Anaplasma marginale*, and *Coxiella burnetii*. Cattle were the most frequently studied host species and exhibited the highest number of pathogen detections, emphasizing their central role in transmission cycles. Wildlife species such as buffalo, impala, and warthogs also contributed to pathogen maintenance and spillover risks.

The review identifies essential gaps in geographic coverage, particularly in Central and West Africa, where limited data hinder a comprehensive understanding. To address these challenges, improved diagnostic capacity, consistent surveillance efforts, and species-specific tick control strategies are needed. Adopting a One Health approach that integrates veterinary, ecological, and public health perspectives will reduce the burden of tick-borne diseases and protect animal and human health across diverse African ecosystems.

## Figures and Tables

**Table 1:** Summary of database search strategies, including search strings, initial search counts, reasons for the reduction, and final imported counts for the systematic review of ticks and tick-borne diseases at the African wildlife–livestock interface.

Database	Search Count	Search String	Reason for Reduction	Imported Count
**Google Scholar**	431	TITLE: ((tick* OR Ixodidae OR “hard tick*”) AND “wildlife–livestock interface” AND (prevalence OR distribution OR “tick-borne disease”))	Irrelevant, language, and outside Africa	23
**PubMed/MEDLINE**	14	((tick* OR ticks OR Ixodidae OR “hard tick*”) AND “wildlife–livestock interface”)	None	14
**Scopus**	24	TITLE-ABS-KEY (ticks OR hard AND ticks AND wildlife–livestock AND interface) AND PUBYEAR > 2014 AND PUBYEAR < 2026	Years	23
**Core**	319	(tick* OR Ixodidae OR “hard tick”) AND “wildlife–livestock interface” AND (prevalence OR distribution OR “tick-borne disease”)	Language and years	298
**Dimension AI**	14	((tick* OR Ixodidae OR “hard tick”) AND “wildlife–livestock interface” AND (prevalence OR distribution OR “tick-borne disease”)), 2025 or 2023 or 2021 or 2020 or 2018 or 2017 or 2016, Article	None	13
**Lens**	53	Scholarly Works (53) = ((tick* OR (Ixodidae OR “hard tick”)) AND (“wildlife–livestock interface” AND (prevalence OR (distribution OR “tick-borne disease”))))	None	53
**Science Direct**	124	tick AND wildlife AND livestock AND interface	None	124

**Table 2:** Characteristics of all eligible studies reporting the distribution and prevalence of ticks and tick-borne pathogens at the wildlife–livestock interface in Africa.

Country/Region	Hosts	Sample Size	Total Pathogens	Pathogens with Prevalence	Ref
Queen Elizabeth NP, Uganda	Cattle	208	6	*Theileria* spp.—50.5% *Theileria parva*—27.9% *Anaplasma marginale*—19.2% *Anaplasma platys-like*—11.5% *Babesia bigemina*—8.7% *Theileria velifera* and *T. mutans*—detected (via sequencing), prevalence not quantified	[16]
Serengeti NP, Tanzania	Cattle	770	1	*Theileria parva*—5.07% (95% Confidence Interval: 3.70–7.00%)	[28]
Budongo Forest, Uganda	Goats (Caprines), Sheep (Ovines)	712 (666 goats, 46 sheep)	6	*Anaplasma* spp.—3.65% (microscopy) *Theileria* spp.—0.42% (microscopy) *Trypanosoma* spp.—0.56% (microscopy) *Trypanosoma brucei*—5.76% (PCR) *Trypanosoma congolense* (Kilifi strain)—0.14% (PCR) Mixed *Trypanosoma* infections—0.98% *T. b. rhodesiense* (detected via SRA gene)—0.14%	[23]
Zimbabwe (south-eastern lowveld—Gonarezhou NP, Malilangwe Conservancy)	Goats	563	2	*Chlamydia abortus*—22.0% overall Porous interface: 21.1% Non-porous interface: 36.8% Non-interface: 9.2% *Brucella* spp.—detected, prevalence not quantified	[34]
Mikumi NP, Tanzania	Cattle	252 cattle, 630 ticks collected	0	N/A—The study identified tick species	[42]
South Africa (Mnisi community—bordering Kruger National Park, Mpumalanga)	Cattle	184	5	*Coxiella burnetii*—38.0% *Toxoplasma gondii*—33.2% *Chlamydophila abortus*—20.7% *Neospora caninum*—1.6% *Rift Valley fever virus* (RVFV)—0.5%	[22]
Tanzania (Mikumi National Park interface)	Ticks	400 ticks	17+ virus families (metagenomic detection)	*Retroviridae*—60.0% *Caulimoviridae*—5.9% *Rhabdoviridae*—5.4% *Herelleviridae*—4.8% Unclassified +ssRNA viruses—3.8% *Mimiviridae*—2.7% *Siphoviridae*—2.6% *Totiviridae*—2.2% Other detected families (1–2%): *Iridoviridae*, *Orthomyxoviridae*, *Chuviridae*, *Phenuiviridae*, *Parvoviridae*, *Podoviridae*, *Myoviridae*	[43]
Botswana (Northern—Maun West & Chobe West)	Cattle	301	4	*Anaplasma* spp.—90.0% *Babesia bigemina*—29.5% *Babesia bovis*—9.2% *Theileria parva*—2.4%	[13]
Kenya (Lambwe Valley—Ruma National Park interface)	Cattle (zebu)	680	6	*Anaplasma bovis*—17.4% *Anaplasma platys* clade—16.9% *Anaplasma marginale*—0.6% *Anaplasma* sp. *Lambwe-1*—11.6% *Theileria velifera*—40.0% *Theileria mutans*—25.7%	[44]
Kenya (Laikipia and Maasai Mara National Reserve)	Wildlife, ticks	Wildlife: 152 Ticks: 851 (Laikipia: 756; MMNR: 95)	1	*C. burnetii* Wildlife (Laikipia & MMNR): Negative Ticks (Laikipia): 2.92% of pools positive (max likelihood individual prevalence: 0.54%) Positive tick species: *Rhipicephalus appendiculatus*, *R. pulchellus*, *R. evertsi* Ticks from MMNR: Negative	[45]
Bushbuckridge, South Africa	Humans	138	4	*Bartonella* spp.—9.5% Spotted Fever Group (SFG) *Rickettsia*—24.1–63.4% *Coxiella burnetii*—2.3–37.8% *Leptospira* spp.—6.8%	[46]
Mozambique (Gorongosa National Park and buffer zone)	Soft ticks	1865 soft ticks	1	*African Swine Fever Virus* (ASFV) PCR-positive pools: 18.8% (Gorongosa NP), 15.4% (buffer zone) Virus isolation: 47.4% of PCR-positive samples Genotypes identified: – Genotype II (11 isolates) – Genotype V (3 isolates) – Novel Genotype XXIV (5 isolates)	[47]
Tanzania (Northern—Simanjiro and Monduli interface areas)	Cattle, Buffalo	Cattle: 160 Buffalo: 22	1	*Theileria parva* 10.0% of cattle carried buffalo-derived *T. parva* (p67 allele 4) 85.0% of cattle carried cattle-type allele 1 Buffalo harbored p67 alleles 2 and 3	[38]
Tanzania (Mikumi National Park interface, Morogoro Region)	Cattle, Goats, Environment	436 animals (260 cattle, 176 goats) 847 ticks collected	0	Not assessed—tick diversity study	[48]
Zimbabwe	Cattle, African buffalo, kudu, impala	Cattle: 1011 Buffalo: 111 Kudu: 18 Impala: 32	1	*Brucella* spp. Cattle: 16.7% overall – Porous interface: 19.5% – Non-porous interface: 15.4% – Non-interface: 13.0% African buffalo: 20.7% overall – Mabalauta: 30.4% – Chipinda Pools: 18.2% Kudu & Impala: 0% (not detected)	[40]
Kenya (Narok County—Maasai Mara wildlife–livestock interface)	Cattle, Sheep, Wildebeest	61 animals (30 cattle, 30 wildebeest, 1 sheep); 165 ticks collected	5	*Theileria parva*—detected in 18 ticks *Theileria equi*—detected in 4 ticks *Theileria ovis*—detected in 1 tick *Babesia microti*-like—detected (strain not quantified) *Babesia canis*-like—detected (strain not quantified)	[49]
Kenya (Laikipia and Maasai Mara ecosystems)	Wildlife; Ticks	152 wildlife 851 ticks	2	Spotted Fever Group *Rickettsia* spp. Wildlife: 2.5% (Laikipia), 5.5% (Maasai Mara) Ticks: 21.9% (Laikipia), 17.2% (Maasai Mara) Species identified: *Rickettsia sibirica*, *R. sibirica mongolotimonae* *Coxiella burnetii* (Q fever) Ticks (Laikipia): 2.9% Positive tick species: *Rhipicephalus appendiculatus*, *R. pulchellus*, *R. evertsi evertsi*	[33]
Kenya (Various wildlife–livestock interface zones: Nairobi NP, Maasai Mara, Laikipia, Marula)	Lions, Leopards, Cheetahs, Giraffes, Waterbuck	253	2	*Theileria* spp.—22.3% to 34.6% *Babesia* spp.—8.2% to 19.4%	[50]
Kenya (Amboseli ecosystem, Kajiado County)	Impalas, Sheep, Goats	Sheep: 200 Goats: 300 Impalas: 20	1	*Coxiella burnetii* (Q fever) Impalas—25.0% Goats—21.7% Sheep—6.0%	[39]
Zimbabwe (Southeast lowveld—Gonarezhou and Kruger NP interface)	Cattle, Wildlife (buffalo, impala, kudu, giraffe)	Cattle: 1158 Wildlife: 97	1	*Brucella* spp. Cattle: 9.9% overall – Interface areas: 10.3% – Non-interface areas: 6.7% – Grazing in park: 13.5% vs. 4.9% (not grazing) – Females with abortion history: 70.7% vs. 6.3% (no history) Wildlife: 1.03%—detected in one giraffe only	[51]

## Data Availability

No new data were created or analyzed in this study. Data sharing is not applicable to this article.

## Appendix A

**Table A1:** PRISMA 2020 Main Checklist.

Topic	No.	Item	Location Where Item Is Reported
**TITLE**			
**Title**	1	Identify the report as a systematic review.	Title: “A Systematic Review” is included
**ABSTRACT**			
**Abstract**	2	See the PRISMA 2020 for Abstracts checklist	
**INTRODUCTION**			
**Rationale**	3	Describe the rationale for the review in the context of existing knowledge.	Introduction: Provides background and justification
**Objectives**	4	Provide an explicit statement of the objective(s) or question(s) the review addresses.	Introduction: Clearly states the research question
**METHODS**			
**Eligibility criteria**	5	Specify the inclusion and exclusion criteria for the review and how studies were grouped for the syntheses.	Section 2
**Information sources**	6	Specify all databases, registers, websites, organisations, reference lists and other sources searched or consulted to identify studies. Specify the date when each source was last searched or consulted.	Section 2.3
**Search strategy**	7	Present the full search strategies for all databases, registers, and websites, including any filters and limits used.	Section 2.2
**Selection process**	8	Specify the methods used to decide whether a study met the inclusion criteria of the review, including how many reviewers screened each record and each report retrieved, whether they worked independently, and if applicable, details of automation tools used in the process.	Table 1—Summary of database search strategies
**Data collection process**	9	Specify the methods used to collect data from reports, including how many reviewers collected data from each report, whether they worked independently, any processes for obtaining or confirming data from study investigators, and if applicable, details of automation tools used in the process.	Section 2.4
**Data items**	10a	List and define all outcomes for which data were sought. Specify whether all results that were compatible with each outcome domain in each study were sought (e.g., for all measures, time points, analyses), and if not, the methods used to decide which results to collect.	Section 2.4
	10b	List and define all other variables for which data were sought (e.g., participant and intervention characteristics, funding sources). Describe any assumptions made about any missing or unclear information.	Section 2.4
**Study risk of bias assessment**	11	Specify the methods used to assess risk of bias in the included studies, including details of the tool(s) used, how many reviewers assessed each study and whether they worked independently, and if applicable, details of automation tools used in the process.	Risk of Bias Assessment section
**Effect measures**	12	Specify for each outcome the effect measure(s) (e.g., risk ratio, mean difference) used in the synthesis or presentation of results.	Section 3
**Synthesis methods**	13a	Describe the processes used to decide which studies were eligible for each synthesis (e.g., tabulating the study intervention characteristics and comparing against the planned groups for each synthesis (item 5)).	Synthesis Methods section, Figure 3, Figure 4, Figure 5, Figure 6, Figure 7, Figure 8, Figure 9 and Figure 10
	13b	Describe any methods required to prepare the data for presentation or synthesis, such as handling of missing summary statistics, or data conversions.	Synthesis Methods section
	13c	Describe any methods used to tabulate or visually display results of individual studies and syntheses.	Tables and Figures
	13d	Describe any methods used to synthesize results and provide a rationale for the choice(s). If meta-analysis was performed, describe the model(s), method(s) to identify the presence and extent of statistical heterogeneity, and software package(s) used.	Synthesis Methods section, Figure 3, Figure 4, Figure 5, Figure 6, Figure 7, Figure 8, Figure 9 and Figure 10
	13e	Describe any methods used to explore possible causes of heterogeneity among study results (e.g., subgroup analysis, meta-regression).	Section 3
	13f	Describe any sensitivity analyses conducted to assess robustness of the synthesized results.	Section 3
**Reporting bias assessment**	14	Describe any methods used to assess risk of bias due to missing results in a synthesis (arising from reporting biases).	Risk of Bias Assessment section
**Certainty assessment**	15	Describe any methods used to assess certainty (or confidence) in the body of evidence for an outcome.	Risk of Bias Assessment section
**RESULTS**			
**Study selection**	16a	Describe the results of the search and selection process, from the number of records identified in the search to the number of studies included in the review, ideally using a flow diagram.	PRISMA Flow Diagram (Figure 2)
	16b	Cite studies that might appear to meet the inclusion criteria, but which were excluded, and explain why they were excluded.	Section 2.5
**Study characteristics**	17	Cite each included study and present its characteristics.	Table 2—Characteristics of included studies
**Risk of bias in studies**	18	Present assessments of risk of bias for each included study.	Risk of Bias Assessment section (Figure 11)
**Results of individual studies**	19	For all outcomes, present, for each study: (a) summary statistics for each group (where appropriate) and (b) an effect estimate and its precision (e.g., confidence/credible interval), ideally using structured tables or plots.	Tables and Figures (e.g., prevalence per tick species)
**Results of syntheses**	20a	For each synthesis, briefly summarise the characteristics and risk of bias among contributing studies.	Figure 3, Figure 4, Figure 5, Figure 6, Figure 7, Figure 8, Figure 9 and Figure 10
	20b	Present results of all statistical syntheses conducted. If meta-analysis was conducted, present for each the summary estimate and its precision (e.g., confidence/credible interval) and measures of statistical heterogeneity. If comparing groups, describe the direction of the effect.	Figure 3, Figure 4, Figure 5, Figure 6, Figure 7, Figure 8, Figure 9 and Figure 10
	20c	Present results of all investigations of possible causes of heterogeneity among study results.	Section 3
	20d	Present results of all sensitivity analyses conducted to assess the robustness of the synthesized results.	Section 3
**Reporting biases**	21	Present assessments of risk of bias due to missing results (arising from reporting biases) for each synthesis assessed.	Risk of Bias section
**Certainty of evidence**	22	Present assessments of certainty (or confidence) in the body of evidence for each outcome assessed.	Risk of Bias section
**DISCUSSION**			
**Discussion**	23a	Provide a general interpretation of the results in the context of other evidence.	Section 4
	23b	Discuss any limitations of the evidence included in the review.	Discussion—Regional and Surveillance Gaps
	23c	Discuss any limitations of the review processes used.	Discussion—Screening and Selection Process Limitations
	23d	Discuss implications of the results for practice, policy, and future research.	Discussion—Future Directions and One Health Approach
**OTHER INFORMATION**			
**Registration and protocol**	24a	Provide registration information for the review, including register name and registration number, or state that the review was not registered.	The review was not registered.
	24b	Indicate where the review protocol can be accessed, or state that a protocol was not prepared.	A protocol was not prepared.
	24c	Describe and explain any amendments to information provided at registration or in the protocol.	Not applicable
**Support**	25	Describe sources of financial or non-financial support for the review, and the role of the funders or sponsors in the review.	Not applicable
**Competing interests**	26	Declare any competing interests of review authors.	Conflicts of Interest section
**Availability of data, code and other materials**	27	Report which of the following are publicly available and where they can be found: template data collection forms; data extracted from included studies; data used for all analyses; analytic code; any other materials used in the review.	Data Availability Statement

## Appendix B

**Table A2:** PRIMSA Abstract Checklist.

Topic	No.	Item	Reported?
**TITLE**			
**Title**	1	Identify the report as a systematic review.	Yes
**BACKGROUND**			
**Objectives**	2	Provide an explicit statement of the main objective(s) or question(s) the review addresses.	Yes
**METHODS**			
**Eligibility criteria**	3	Specify the inclusion and exclusion criteria for the review.	Yes
**Information sources**	4	Specify the information sources (e.g., databases, registers) used to identify studies and the date when each was last searched.	Yes
**Risk of bias**	5	Specify the methods used to assess risk of bias in the included studies.	Yes
**Synthesis of results**	6	Specify the methods used to present and synthesize results.	Yes
**RESULTS**			
**Included studies**	7	Give the total number of included studies and participants and summarise relevant characteristics of studies.	Yes
**Synthesis of results**	8	Present results for main outcomes, preferably indicating the number of included studies and participants for each. If meta-analysis was conducted, report the summary estimate and confidence/credible interval. If comparing groups, indicate the direction of the effect (i.e., which group is favoured).	Yes
**DISCUSSION**			
**Limitations of evidence**	9	Provide a brief summary of the limitations of the evidence included in the review (e.g., study risk of bias, inconsistency and imprecision).	Yes
**Interpretation**	10	Provide a general interpretation of the results and important implications.	Yes
**OTHER**			
**Funding**	11	Specify the primary source of funding for the review.	Yes
**Registration**	12	Provide the register name and registration number.	No

*From:* Page [81].
